# Advancing health equity by addressing social determinants of health: Using health data to improve educational outcomes

**DOI:** 10.1371/journal.pone.0247909

**Published:** 2021-03-17

**Authors:** Mary Jo Rattermann, Azure Angelov, Tommy Reddicks, Jess Monk

**Affiliations:** 1 Paramount Health Data Project, Indianapolis, Indiana, United States of America; 2 Paramount Schools of Excellence, Indianapolis, Indiana, United States of America; George Washington University, UNITED STATES

## Abstract

Data from two social determinants of health—access to health care and access to a quality education—are combined to examine the impact of health on student achievement. Data from a high poverty, high performing K-8 school revealed a significant negative correlation between the number of visits to a school-based nurse and standardized academic assessments. Fixed effect regression confirmed the effect of total number of visits to the school-based nurse on performance on standardized assessments, and also revealed that two types of visits, neurological and gastrointestinal, were predictive of student performance. Taken together, these results suggest that when students are suffering from ill health their academic performance is negatively impacted. Implications for improving health equity through data-driven educational interventions are discussed.

## Introduction

Health equity means that everyone has a fair and just opportunity to be as healthy as possible [1]. To achieve health equity, access to the conditions and resources that positively influence health must be available to everyone, including historically excluded or marginalized groups of society. These conditions and resources that influence health are often referred to as the *social determinants of health*. Specifically, social determinants of health are conditions in the environment in which people live, learn, work, play, worship, and age that affect a wide range of health, functioning, and quality of life outcomes and risks. Examples of social determinants of health include quality of education, public safety, access to health care services, social support, residential segregation, among others [2].

These social determinants of health often interact with one another to create complex systems that influence long-term outcomes [3, 4]. Two such factors that have a significant impact on children of poverty in the United States are access to health care and quality of education. Equitable access to quality education in the United States is one of the areas that has the most striking disparities based on socio-economic status. Economic segregation, as measured by how evenly poor and nonpoor students are distributed among public schools, has been on the increase [5], with students of poverty being more likely to be in schools that lack access to the high-quality resources and learning opportunities that are often available to their wealthier peers [6, 7]. For example, teachers at high poverty schools have less experience than teachers in low poverty schools [8, 9] and often have fewer formal qualifications and have less resources to use during classroom instruction [10]. This inequity directly impacts student academic achievement, with students in high-poverty/high-minority schools consistently performing poorer in measures of academic success than white students in low-poverty schools [7, 11].

Access to quality health care is also affected by socioeconomic status. Despite the great gains made in access to healthcare since the passage of the Affordable Care Act, children from economically disadvantaged communities often lag behind their peers in more affluent communities in access to quality health care [12–14]. Over one quarter (26.3%) of poor children under 18 years of age in the United States have gone more than 6 months without having contact with a doctor or other health care professional, with 4.2% of those children having no contact with a doctor or health care professional in over five years [15].

Many schools are addressing the lack of access to healthcare through school nurse programs and school-based health centers. School-based health care provides access to primary and preventive health care services for students who are at risk for negative medical, social and academic outcomes [16, 17]. For children of poverty, the school nurse and the school-based health center are often the only medical care that is available to them [18, 19]. School based health care also has the added benefit of being located where children spend large periods of time–often 8 or more hours a day and over 180 days per year. Consequently, there is no need for parents to provide transportation to the location of the health provider, or for them to take time off work to accompany their child to the doctor [20–22] leading to more frequent access to health care [23, 24]. Additionally, school nurses and school-based health centers are often located in schools that serve students of poverty who are more likely to be chronically stressed, tired, hungry, and have vision and hearing problems, all factors that put a student at risk for academic failure [25–27]. School-based health care has been shown to have a positive effect on students, both in their academic behaviors such as attendance, suspensions, withdrawals from school and graduation rates [28–31] and in their social and emotional well-being, with a positive effect on school connectedness [32–35] and on quality of life [36].

The goal of this research is to examine the relationship between two social determinants of health: access to health care and quality education. The data for this research comes from a high-performing K-8 charter school that serves a high poverty community with a large minority population; 65% of the students in the school are African-American or Hispanic and 83%are children of poverty (as measured by free and reduced lunch). The primary hypothesis of this research is that academic achievement, as measured by standardized test scores, will be impacted by overall student health, as measured by the number of visits to a school nurse. A secondary hypothesis is that the reason for a visit to the school nurse, not just the number of visits, will also have an impact. Specifically, that respiratory complaints or complaints of parasites and skin rashes, both of which previous research has found to be related to poverty, [37, 38] will be found to impact performance on measures of academic achievement.

## Materials and methods

### Ethics statement

Prior to commencement of the study, ethical approval (IRB No.2) was obtained from the Institutional Review Board (IRB) of IntegReview IRB. The research was determined to be a clinical investigation not involving greater than minimal risk under an expedited review. Assent was waived under 45 CFR, 46.116(d) due to the use of retrospective data that were deidentified for this research.

## Participants

The data used in these analyses are from the Paramount School of Excellence Brookside (Paramount Brookside), which is a public charter school on the near eastside of Indianapolis. Paramount Brookside serves urban students in grades K-8. With a diverse student body of more than 1000 students, Paramount Brookside provides a sample of students who are living in poverty—with 83% of students qualifying for free and reduced meals, a common indicator of poverty. The students at Paramount Brookside are also ethnically diverse, with African American students making up 49% of Paramount Brookside’s population, while Latinos are 16%, Multiracial students are 10%, and European American students are 25%. Almost 20% of PSOE students are identified as students with disabilities and receive special education services and 6% are identified as English Language Learners. Paramount Brookside is a high performing school, with approximately 90% of all Paramount Brookside students passing the state mandated IREAD 3 test and more than 80% of all Paramount Brookside students passing both the English and Math portions of the state mandated ISTEP test during the years corresponding to the data being used in this research.

To examine the questions posed in this study a dataset was constructed that included student demographics such as gender, race/ethnicity and grade, the number of visits to the school nurse, and student scores on Acuity, a standardized assessment of student academic achievement. The Acuity assessment is administered longitudinally at three time points of the year (i.e., Acuity A is given at the beginning of the academic year, Acuity B is given in the middle, and Acuity C is given near the end) in two subject areas: Mathematics and English Language Arts (ELA).

### Data collection method

Since 2013 Paramount Brookside has employed a full-time nurse, who during the time of the data collection saw 70–100 students per day. These visits range from a student who is sick with the flu to a student who find the class overwhelming and simply needs to talk to a sympathetic adult. The data were entered into the University of Tennessee’s Consortium for Health Education, Economic Empowerment, and Research (CHEER) data collection platform, following the CHEER protocols for all visits to the nurse’s office. The student health data and the academic achievement data were deidentified and merged by an independent third party acting as an honest broker of the data. All visits to the school nurse for either administration of prescription or over the counter drugs were removed from the dataset. All reported analyses were performed on the deidentified dataset.

### Categorization of variables

At the time of a student visit to the school nurse, the student characteristics (e.g., grade, gender and race/ethnicity) were recorded using the CHEER data collection platform. The school nurse also recorded the reason for the student’s visit and the outcome of that visit (e.g., the student was sent back to class, was sent home, etc.). The school nurse categorized the reason for the student visit into one of 16 categories (excluding regular prescription and over the counter medication administrations) from the CHEER protocol: (1) Cardiovascular (e.g., blood pressure check, chest pain), (2) Dental (e.g., toothache, lost tooth), (3) Dermatological (e.g., rash, eczema, bug bite, splinter), (4) Eye/Ears/Nose/Throat (e.g., earache, sore throat, pinkeye), (5) Endocrine (e.g., blood sugar check), (6) Gastrointestinal (e.g., heartburn, nausea, diarrhea), (7) Genitourinary (e.g., urinary frequency, soiled clothing), (8) Gynecological/Obstetrical (e.g., menstrual cramps), (9) Musculoskeletal (e.g., body aches, swelling), (10) Nutrition/Metabolic (G tube feed), (11) Neurological (e.g., headache, dizziness, falling asleep in class), (12) Parasites/Infections (e.g., bed bugs, head lice, ticks), (13) Disorder from Physical Agents (e.g., electrical shock), (14) Psychosocial (e.g., anxiety, altercation, tearful, needs to talk), (15) Respiratory (e.g., asthma, choking, cough, shortness of breath), (16) Other/Miscellaneous (e.g., physical injury, chills, illness).

The data from the 2013–14, 2015–16 and 2016–17 school years were included in these analyses. The data from the 2014–2015 school year were not included due to the lack of student achievement data for that year. 584 students from grade 3–8 contributed 933 sets of data (one year of test scores and one year of health data) to this dataset. Of the 933 sets of data, 48% (or 452) were contributed by girls and 52% (481) were contributed by boys. The majority of the datasets were contributed by African-American students (45% or 419 datasets), followed by white students (29% or 272 datasets), Hispanic students (17% or 157 datasets) and then multiracial students (9% or 86 datasets). Each of these students provided at least one year of Acuity data. Overall, these students visited the nurse’s office a total of 4467 times over the 3 years of the data being used in this study. The most common frequency of nurse office visits per student was "none” for 21% of students, followed by 1–4 for 42% of students, and 5–10 for 30% of students. The remaining 7% of students visited the school nurse office from 11–84 times.

## Results

The quantitative data were analyzed using SPSS 26.0 (IBM Corp., Armonk, NY, USA). Participants’ general characteristics were analyzed with descriptive statistics, significant differences between these general characteristics were analyzed with ANOVA. Post hoc analyses were done using the Bonferroni adjustment to correct for multiple comparisons. For correlations between the number of visits to the school nurse and educational outcomes, Pearson’s correlation coefficients were computed.

### Results by demographics

Across the three school years, male students had slightly more yearly visits to the nurse (51%) as compared to female students (49%), with the mean number of visits to the school nurse per year for female students (5.31) and male students (5.17) roughly equal. African-American students accounted for the majority of the total nurse visits (50%) as compared to 32% for white students, 10% for Hispanic students and 8% for multiracial students. The mean number of visits to the school nurse was 5.83 for African-American students, 5.72 for White students, 3.29 for Hispanic students and 4.31 for Multiracial students.

Visit frequencies of students across grades varied significantly, with younger students accounting for more visits to the school nurse than older students. Specifically, students in grades 3 and 4 both accounted for 21% of visits to the school nurse, 5th grade students accounted for 18%, 6th grade students accounted for 16%, 7th grade students accounted for 14% and 8th grade students accounted for 9%. The mean number of visits by grade revealed that 3rd grade students visited the school nurse an average of 5.11 times, 4th grade students visited the school nurse an average of 6.38 times, 5th grade students visited an average of 5.55 times, 6th grade students visited an average of 5.39 times, 7th grade students visited an average of 3.52 times, and finally 8th grade students visited an average of 4.59 times.

These findings were confirmed by a race (Black, White, Hispanic and Multiracial) Gender (male and female) x Grade (3rd, 4th, 5th, 6th, 7th and 8th grades) within subjects Analysis of Variance performed on the total number of visits to the school nurse which found that Race was significant (F(3,884) = 4.54, p < .01) as was Grade (F(3,88 5) = 4.54, p < .004). There were no other main effects and no significant interactions. Bonferroni post hoc analyses revealed that the mean number of visits to the school nurse by African American (5.83) and White students (5.72) were not significantly different from each other but were significantly different from the mean number of visits to the school nurse by Hispanic (3.29) and Multiracial students (4.3) who were not significantly different from each other. Post hocs revealed a significant difference between the mean number of visits to the school nurse between 4*th* grade students (6.38) and 7*th* grade students (3.52).

### Correlation between health data and academic data

The primary hypothesis tested in this research is that the number of visits to the school nurse is predictive of student achievement scores. To determine the effect of the number and reason for visits to the school nurse office a series of bivariate correlations were performed over the 16 categories of health reasons and the three yearly Acuity scores for Language Arts and Mathematics. A conservative *α* value of 0.01 was used to address the possibility of finding a significant result by chance alone because of multiple comparisons.

Pearson correlation analyses investigating the impact of the type and number of visits to the school nurse on academic achievement revealed a negative correlation between total number of visits and scores on the Acuity tests across time in Language Arts, with the comparison between the first administration in a school year of Language Arts Acuity A revealing a significant correlation (-.196) between the total number of visits to the school nurse and the Acuity scores. This relationship was also found in the second administration in a school year of Language Arts Acuity B, with the comparison between the second administration and the total number of visits to the school nurse also revealing a significant correlation (-.174). A weaker, but still significant correlation was also found between the third administration of Language Arts Acuity C (-.118). Similar results were found in Acuity Mathematics, although the correlations were not as strong as those with Language Arts. All of the significant correlations can be found in Table 1 below.

**Table 1:** Correlations between health data and academic achievement.

	ELA Acuity A	ELA Acuity B	ELA Acuity C	Math Acuity A	Math Acuity B	Math Acuity C
Total	-.196**	-.174**	-.118**	-.096**	-.074	-.063
Neurological	-.121**	-.099**	-.094**	-.074	-.049	-.057
Gastrointestinal	-.122**	-.095**	-.061	-.057	-.015	-.031
Dermatological	-.152**	-.156**	-.063	-.059	-.071	-.040
Musculoskeletal	-.130**	-.139**	-.098**	-.057	-.068	-.056
Eyes, Ears, Nose	-.127**	-.129**	-.060	-.033	-.012	.007
Other	-.117**	-.114**	-.085**	-.087**	-.089**	-.063

In addition to significant correlations between the total number of visits to the school nurse, signification correlations were found between Language Arts Acuity and Neurological, Gastrointestinal, Dermatological, Musculoskeletal, Eyes, Ears, Nose & Throat and “other” types of visits to the school nurse. These correlations were particularly strong for the first and second administration of Language Arts Acuity.

To further explore the relationship between the number and type of visits to the school nurse and academic achievement as measured by Acuity a series of fixed effects regressions, or linear regressions with unit and time fixed effects, were performed on the longitudinal academic assessment data. The use of a fixed effects regression also adjusted for unobserved unit-specific and time-specific confounds that may have been present in the data.

Because the data from the 2014–15 school year were not available, only the data from the 2015–16 and 2016–17 school years were contiguous. Consequently, these analyses were performed over a subset of the data that included only student data from those years. This decreased the dataset from 584 to 155 students. In this new dataset students from grade 3–8 contributed 155 sets of data (two year of test scores and two years of health data each). Of the 155 sets of data, 45% (or 141) were contributed by girls and 55% (169) were contributed by boys. The majority of the datasets were contributed by African-American students (48% or 149 datasets), followed by white students (25% or 79 datasets), Hispanic students (17% or 54 datasets) and multiracial students (9% or 28 datasets).

The fixed effects regressions were performed with *student* as the unit variable and *year (1 and 2)* as the time variable. The outcome variable for these analyses was student *Growth Scores* for the Acuity English Language Arts and Mathematics assessments. Growth Scores are computed by subtracting student scores on the first Acuity administration in each academic year (Acuity A) from the final administration in each academic year (Acuity C) in each area (English Language Art and Mathematics). As a Student Growth Assessment, Acuity is designed to be used to determine student growth over the course of the academic year. Because the independent variable in these analyses (number/type of visits to the school nurse) is cumulative for the school year, student academic growth measured at the end of the academic year was determined to be the appropriate outcome variable.

The quantitative data were analyzed using Stata/IC 16.1 for Mac (StataCorp, College Station, TX, USA). All of the fixed effects (within) regressions were performed with *student* as the Panel variable and *Year (1 and 2)* as the Time variable. Hausman tests for random effects versus fixed effects models were performed for each of the significant analyses reported and in each case the null hypothesis was rejected, confirming that a fixed effects model was appropriate.

The Language Arts Acuity Growth analyses revealed significant effects of Total Number of Visits (F(1,154) = 6.38, p < .01), Dermatological visits (F(1,154) = 4.53, p < .05), and Gastrointestinal visits, (F(1,154) = 3.73, p < .05). There were no significant effects revealed in the Mathematics Acuity Growth analyses.

### Types of visits to the school nurse

The most common type of visits to the school nurse were for Gastrointestinal complaints (e.g., upset stomach, gastric reflux) and Ear, Nose and Throat (e.g., sore throats) which both accounted for 20% of the visits to the school nurse. Dermatological (e.g., rashes) at 16% of the total and Neurological (headaches) at 13% of the total were the two next most common complaints. The frequency and type of visits to the school nurse can be found in Table 2 below.

**Table 2:** Frequency of types of visits to the school nurse.

Gastrointestinal	20%
Eyes, Ears, Nose, Throat	20%
Dermatological	16%
Musculoskeletal	13%
Neurological	13%
Respiratory	6%
Gynecological	5%
Dental	5%
Parasites	1%
Psychosocial	1%
Cardiovascular	0%
Endocrine	0%
Genitourinary	0%
Nutrition	0%
Physical Agents	0%

## Discussion

The results of this research revealed that there is a significant negative correlation between the number of visits to a school nurse and academic achievement as measured by a longitudinal standardized assessment of academic achievement. This effect was stronger for English Language Arts than for Mathematics, and for English Language Arts this effect was consistent over three testing periods. Further analyses revealed that of the 16 different types of visits to the school nurse, performance in English Language Arts was correlated to neurological, gastrointestinal, dermatological, musculoskeletal, Eyes, Ears, Nose & Throat, and “other” categories of visits types.

Additional fixed effect regression analyses confirmed and expanded the results of the correlations, suggesting that there is a causal relationship between the number and type of visits to the school nurse and academic achievement. The fixed effects regression analyses found significant effects of the total number of visits to the school nurse, as well as the type of visits (dermatological and neurological) to the school nurse on student growth in Acuity scores in English Language Arts.

Overall, the results of this research support our primary hypothesis that academic achievement, as measured by standardized test scores, is impacted by overall student health, as measured by the number of visits to a school nurse. The results also support our secondary hypothesis that the reason for a visit to the school nurse, not just the number of visits, will also have an impact, although the specific category of complaints predicted based on the literature, parasites and respiratory complaints were not the categories found to be significant in this research. Rather, dermatological and gastrointestinal complaints were predictive of academic performance for the sample of students studied here. This particular finding may be due to characteristics of this population and deserves further study. It is also important to note that the categories used by the CHEER protocol are broad and the specific diagnoses (e.g, rash versus bug bite for dermatological and heartburn versus nausea for gastrointestinal) were not available for research and could have yielded more specific conclusions.

There are several possible explanations for the results of this research. First, the data suggests that students who are frequent visitors to the school nurse are simply unhealthy and frequently do not feel well during the school day, and this is impacting their ability to learn. As noted by Basch and his colleagues “healthier students are better learners” [39–41]. Research on the impact of health on academic behaviors supports this argument [39, 40, 42–44]. Children who are unhealthy are at higher risk for dropping out [45], failing classes [46], and grade retention [44], while children who exercise regularly, are at a healthy body weight and have access to healthy diets show increased cognitive abilities and positive academic behaviors [42].

A second possible explanation is that students who frequent the school nurse are missing valuable class time and are consequently performing poorly on the standardized assessments. This explanation may be valid when considering the students who have visited the school nurse multiple time per week, but the majority of students in the sample visited the school nurse under 10 times in an academic year. With the average time spent with a school nurse in this sample being approximately 5 minutes, even with travel time to and from the classroom the amount of instruction time missed is minimal in the context of the larger school year.

What is more likely is that many students who are frequent visitors to the school nurse are exhibiting symptoms of larger stressors in their lives. The data from this research supports this hypothesis, with stress related gastrointestinal and neurological complaints making up nearly one third of the reasons for visits to the school nurse, and gastrointestinal complaints also being one of the two significant categories found by the regression analyses. The most common types of somatic complaints (where students visit the school nurse because of vague, varied and sometimes unsubstantiated complaints) found in the literature are headaches, followed closely by stomachaches [47–51]. These somatic complaints have been found to be related to comorbid mental health problems with children classified as “somatizers” being four times more likely to screen for mental health problems than typical peers [52]. Further, children who exhibit somatic complaints are more often in minority, urban and non-intact and less educated families [52].

This research has demonstrated that there is a significant relationship between student health and academic achievement. Additional research needs to be done to provide more specific information regarding the interactions between student health and performance in school. The current findings, however, are enough to begin the process of using data from one social determinant of health—access to health care—to address another—access to a quality education. Based on the significant negative correlation between visits to the school nurse and scores on academic assessments, Paramount School of Excellence Brookside has begun the process of integrating student health data into Multi-Tiered System of Supports (MTSS) [53]. MTSS is a systemic, continuous-improvement framework in which student data is used to inform decision-making that supports students in their academic and socio-emotional development. Specifically, in the MTSS process student data from standardized assessments are used to determine whether a student’s needs are being met by the Tier 1 interventions that are implemented with all students in the classroom setting or if Tier 2 services, such as small group tutoring, are needed to ensure student success. If the student does not show progress with the Tier 2 interventions, then Tier 3 supports will be provided, often through Special Education or other services. MTSS is most effective when it is an early intervention—the sooner a student receives the MTSS services the more effective they will be. Paramount Brookside is using student health data to trigger the MTSS process much sooner than would normally be the case if the move to Tier 2 was triggered by the results of standardized academic assessment s given in the middle of the year.

The ultimate goal of health equity is that everyone has a fair and just opportunity to be as healthy as possible. Addressing social determinants of health in childhood can lead to positive changes across the lifespan, with access to education impacting yearly mortality rates and length of life in adults [54, 55]. In fact, high school graduates live an average of six years longer than adults with less than a high school education, regardless of gender or racial/ethnicity group [56, 57], and have a higher quality of life, reporting greater well-being, fewer chronic health problems, and fewer common illnesses such as colds, headaches and body aches [58]. The results of this research point the way to better use of student health data in schools, which will lead to both better educational and better health outcomes for everyone.
